# When the Gleam of Obstruction Isn’t Just Stones: A Case Report of Intraductal Papillary Neoplasm of the Bile Duct (IPNB) and the Role of Cholangioscopy in Its Management

**DOI:** 10.7759/cureus.76658

**Published:** 2024-12-31

**Authors:** Fatema Mohamed

**Affiliations:** 1 Gastroenterology and Hepatology, Aberdeen Royal Infirmary Hospital, Aberdeen, GBR

**Keywords:** biliary diseases, intraductal papillary neoplasm, intraductal papillary neoplasm of bile duct, intraductal papilloma, peroral cholangioscopy

## Abstract

Intraductal papillary neoplasm of the bile duct (IPNB) is a precursor lesion to biliary tract carcinoma. It is characterised by papillary growth within the bile ducts. The diagnosis and management of IPNB are challenging due to its varying presentations and overlapping features with other biliary diseases. We present a case of a 58-year-old male with a history of vomiting, nausea, and upper abdominal discomfort. He also had a history of Gilbert syndrome and hairy cell leukaemia. Imaging studies showed filling defects in the common bile duct (CBD) and a moderate intrahepatic biliary duct prominence. An endoscopic retrograde cholangiopancreatography (ERCP) revealed a soft tissue lesion in the mid-CBD along with significant biliary dilation. Using SpyGlass cholangioscopy, a large polypoid tumour extending from the ampulla was directly visualised, and biopsies confirmed low-grade dysplasia without invasive characteristics. This report highlights the importance of recognising the clinical and radiological features of IPNB and emphasises the role of cholangioscopy in the management of this rare disorder.

## Introduction

Intraductal papillary neoplasm of the bile duct (IPNB) is identified pathologically as a precursor lesion to invasive cholangiocarcinoma (CCA), with around half of IPNBs exhibiting stromal invasion at the time of surgical resection [1]. IPNB may affect any area of the biliary system, and it manifests as exophytic growth that is grossly apparent in a dilated bile duct lumen. Histologically, the tumorous epithelia are villous or papillary, and they include tubular components that cover tiny fibrovascular stalks [1]. Interestingly, IPNB can be classified into four subtypes: intestinal, gastric, pancreatobiliary, and oncocytic. IPNBs are also categorised as low-grade and high-grade based on lining epithelial features [1].

IPNB differs from the intraductal papillary mucinous neoplasm of the pancreas (IPMN) in its higher histological grade, more advanced stage, increased incidence of concomitant invasive malignancy, and unfavourable prognosis. In the Classification of Digestive System Tumours, 5th edition (2019) published by the World Health Organisation (WHO), only the term IPNB was proposed [1]. Cholangitis, biliary blockage, or the development of invasive cancer are possible outcomes in IPNB [1]. The diverse appearance and histological features of IPNB often lead to diagnostic challenges. Cholangioscopy has emerged as a useful tool to help determine the extent of IPNB and plan the surgical options for its management [1].

## Case presentation

A 58-year-old male with a history of Gilbert syndrome and hairy cell leukaemia presented with long-term episodes of upper abdominal pain. He had also experienced nausea and vomiting for a few weeks. There was no history of weight loss. He had undergone cholecystectomy and prostatectomy in 2019 for gallbladder stones and prostate cancer, respectively. He never consumed alcohol. On examination, the patient was jaundiced with epigastric tenderness. There was no palpable mass or hepatosplenomegaly. Blood investigations showed deranged liver enzymes with a cholestatic pattern (Table 1).

**Table 1:** Blood investigations

Blood test	Patient value	Normal range
Albumin	35	35-50 g/L
Total bilirubin	134	<21 µmol/L
Alanine transaminase (ALT)	65	7-56 units/L
Alkaline phosphatase (ALP)	93	44-147 units/L
Gamma-glutamyl transferase (GGT)	217	9-48 units/L

**Figure 1:**
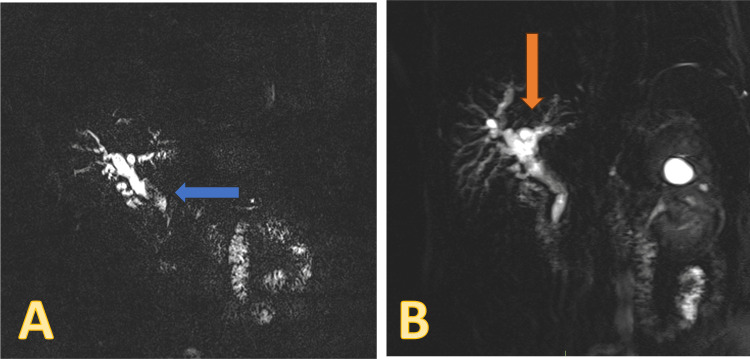
MRCP findings A: bile duct filling defects (blue arrow). B: biliary dilatation disproportionate to post-cholecystectomy status (orange arrow) MRCP: magnetic resonance cholangiopancreatography

An abdominal CT showed a normal liver with mild intrahepatic biliary duct prominence and evidence of previous cholecystectomy Magnetic resonance cholangiopancreatography (MRCP) showed biliary dilatation disproportionate to post-cholecystectomy status with filling defects within the common bile duct, suggestive of stones (Figure 1). 

**Figure 2:**
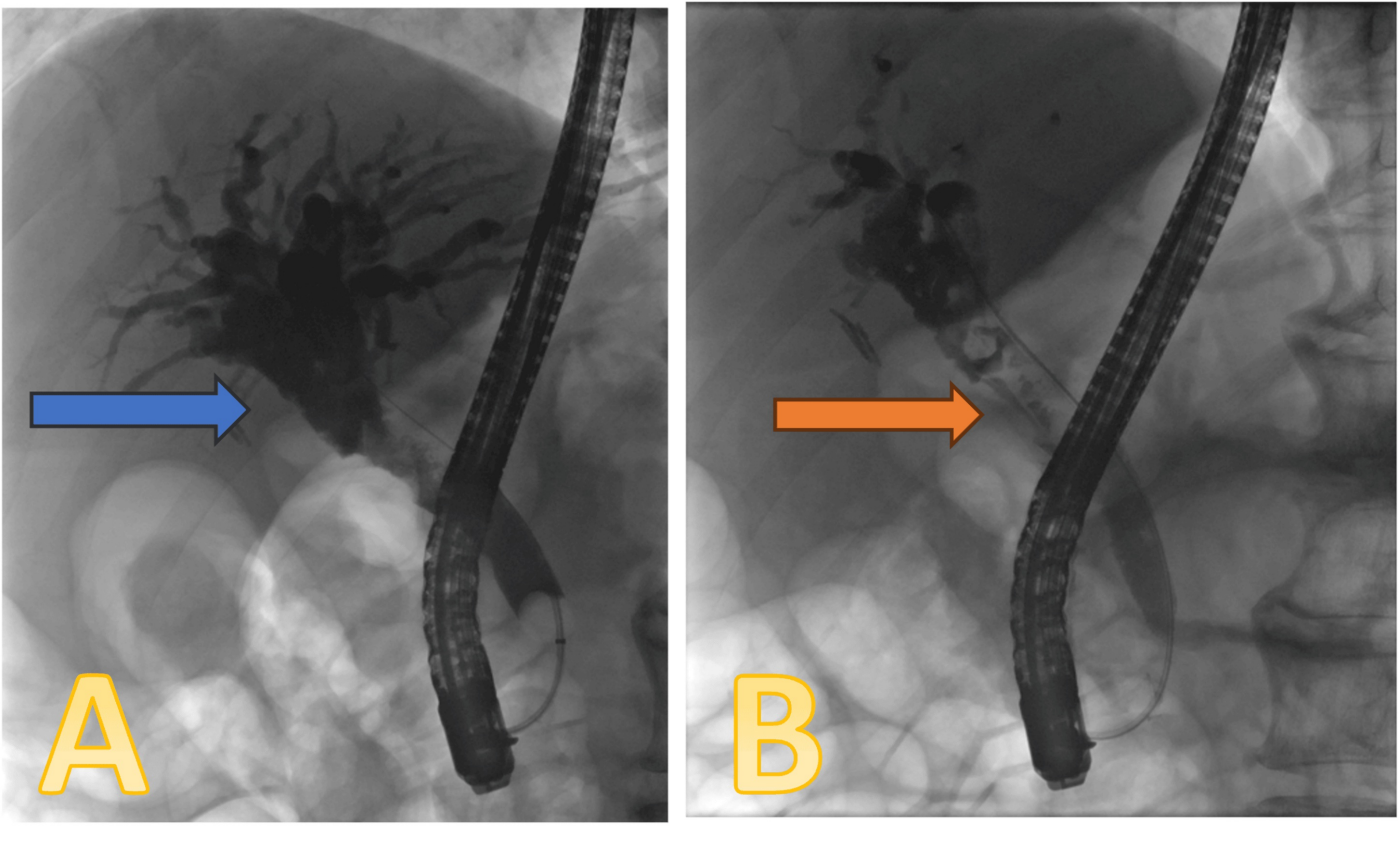
ERCP images A: significant biliary dilation (blue arrow). B: mid-CBD filling defect (orange arrow) CBD: common bile duct; ERCP: endoscopic retrograde cholangiopancreatography

The patient underwent an endoscopic retrograde cholangiopancreatography (ERCP) procedure on November 18, 2023, which showed significant biliary dilation with the suggestion of a soft tissue lesion in the mid-CBD (Figure 2). Sphincterotomy was done, and the duct was trawled with a 12-15-mm balloon. Several small particles of debris and soft tissue were extracted, and brushing samples were taken from the lesion. A 7F x 7-cm double plastic stent was inserted, and a small piece of soft tissue was sent for histology.

The histopathology of the soft tissue obtained during the ERCP showed findings compatible with IPNB with low-grade dysplasia and some atypical and suspicious features. The CT abdomen and pelvis were repeated, and it showed moderate intra- and extrahepatic biliary duct dilation despite a plastic stent appropriately sited within the CBD. Again, there appeared to be a calibre change that was inferior to the insertion of the cystic duct; no obvious masses were identified at this point. The case was discussed at the multidisciplinary team (MDT) meeting, and the plan was to consider cholangioscopy for direct visual evaluation of biliary lesions, which could help diagnose the exact localisation of the lesions and determine the tumour extension for subsequent surgical planning.

**Figure 3:**
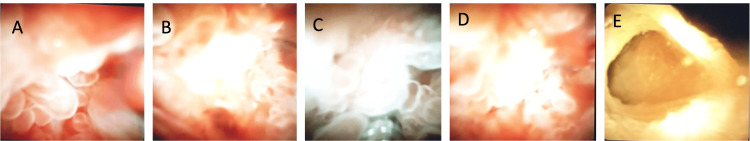
Cholangioscopy images A, B, C, and D: extensive polypoidal papillary lesion starting at 2 cm from the ampulla and extending proximally over 5 cm. E: the hilum

A repeat ERCP was performed on January 24, 2024. The old stent was removed, and a cholangioscope was advanced into the bile ducts up to the hilum of the liver. There was an extensive polypoidal papillary lesion starting at 2 cm from the ampulla and extending proximally over 5 cm, stopping just 1 cm distal to the hilum. The tumour was circumferential in the mid-CBD, but the hilum appeared to be normal and uninvolved. Cholangioscopy-guided biopsies were taken along the lesion, and a 7 x 7 cm plastic double-pigtailed stent was inserted in the bile duct (Figure 3).

The biopsies taken during the ERCP confirmed the diagnosis of IPNB with low-grade dysplasia without any invasive features. The case was discussed at the MDT meeting, and the potential options suggested were surveillance or an operative approach. The operation required would be a Whipple procedure with hepaticojejunostomy. 

## Discussion

IPNB is a rare disease with a prevalence of 4-15% among bile duct tumours [1]. It is a precursor lesion that can develop into cholangiocarcinoma, a bile duct-originating cancer [1]. The tumour cells proliferate intraductally in these neoplasms, causing cystic or papillary lesions in the biliary tree [1]. More than one-third of IPNBs show mucin hypersecretion, and it is more frequently observed in intrahepatic IPNBs than in extrahepatic IPNBs [1]. Bile duct dilation is also more severe in mucin-hypersecreting IPNBs than in mucin-nonsecreting IPNBs [1]. 

Although pancreatic IPMNs and IPNBs share certain histological and clinical aspects, IPNBs have distinctive characteristics and implications for patient management [1]. Approximately half of IPNBs show histopathological features similar to those of IPMNs [1]. However, IPNB differed from IPMN in its higher histological grade, more advanced stage, and worse prognosis [1]. IPNBs are more frequently detected in male patients [2]. The risk factors include cholelithiasis, chronic infections like schistosomiasis [2], primary sclerosing cholangitis, choledochal cysts, and Gardner syndrome [3].

In terms of histology, IPNB may be classified into four subtypes: intestinal, pancreatobiliary, gastric, and oncocytic [1]. Different morphological and immunohistochemical features specific to each subtype may have an impact on treatment options and prognosis management [4]. The intestinal and pancreatobiliary subtypes have a greater likelihood of malignant transformation than the gastric subtype [4]. IPNBs are uncommon, and their vague clinical and laboratory characteristics make the diagnosis more difficult [4]. While silent occurrences are sometimes documented, patients often manifest symptoms such as upper abdominal pain, fever, and jaundice [2]. MRI and CT play a crucial role in diagnosing IPNB before surgery [3]. Significant radiographic characteristics include the presence of bile duct dilatation and intraductal mass [1-3]. The enhancement pattern of IPNB is isodense or hyperdense during the late arterial phase and not hyperdense during the portal-venous and delayed phases [1].

Peroral cholangioscopy (POCS) can visualise the bile duct directly and assess the extent of the tumour [1]. The direct visualisation of the biliary tree is possible by an advanced endoscopic approach, which is not frequently possible with traditional imaging techniques [5]. Clinicians can distinguish IPNBs from other biliary diseases and evaluate the number of lesions with cholangioscopy, which allows them to see specific characteristics including papillary projections, vascular patterns, and mucosal alterations [6]. Furthermore, compared to blind biopsies or brush cytology during the ERCP procedure, cholangioscopy enhances diagnostic accuracy by enabling targeted biopsies of suspicious locations [5]. Direct cholangiography with a biopsy was reported to help determine the extent of IPNB and perform limited surgical resection [1], as it provides precise information about the location, size, and superficial spread of the tumour along the biliary epithelium [6].

It appears that ERCP alone is not as accurate as a direct visual impression during cholangioscopy [7]. To ensure that no minor tumours are left behind and to achieve curative excision, intraoperative cholangioscopy with staged biopsies and frozen sections is recommended [8]. Additionally, cholangioscopy can be helpful in the follow-up of patients with IPNBs, especially those who have undergone partial resections, since it allows for the periodic evaluation of the residual biliary tree to check for new lesions or recurrence [8].

Despite its advantages, cholangioscopy has many limitations. It is an invasive procedure that requires a high degree of skill to perform and interpret. Even though visual impressions obtained during cholangioscopy are quite reliable, a conclusive diagnosis requires a correlation between them and histological results. Because IPNBs frequently include different degrees of cytoarchitectural atypia, pathologic diagnosis by preoperative biopsy cannot always represent the maximal degree of atypia [9].

## Conclusions

IPNBs are a unique type of biliary tract neoplasms. Early detection and proper management are crucial as these lesions tend to become cancerous. Cholangioscopy has emerged as a valuable tool in the management of IPNBs, playing a key role from the initial diagnosis to planning treatment and monitoring. Its capacity for direct visualisation and targeted sampling has significantly enhanced the accuracy of diagnosis and treatment outcomes for patients with rare but potentially malignant biliary lesions. More research is required to enhance diagnostic techniques, perfect treatment methods, and improve long-term results for patients with IPNBs.
